# Dietary Barriers Appear to Influence the Effects of a Dyadic Web-Based Lifestyle Intervention on Caloric Intake and Adiposity: A Mediation Analysis of the DUET Trial

**DOI:** 10.3390/nu15234918

**Published:** 2023-11-25

**Authors:** Harleen Kaur, Gregory Pavela, Dori W. Pekmezi, Laura Q. Rogers, William W. Cole, Kelsey B. Parrish, R. Drew Sayer, Holly R. Wyatt, Wendy Demark-Wahnefried

**Affiliations:** 1 Department of Nutrition Sciences, University of Alabama at Birmingham (UAB), Birmingham, AL 35294, USA; sayerd@uab.edu (R.D.S.); drholly@uab.edu (H.R.W.); demark@uab.edu (W.D.-W.); 2 Department of Health Behavior, University of Alabama at Birmingham (UAB), Birmingham, AL 35294, USA; pavela@uab.edu (G.P.); dpekmezi@uab.edu (D.W.P.); colew14@uab.edu (W.W.C.); kelseyp@uab.edu (K.B.P.); 3 O’Neal Comprehensive Cancer Center at UAB, Birmingham, AL 35233, USA; lqrogers@uabmc.edu; 4 Department of Medicine, University of Alabama at Birmingham (UAB), Birmingham, AL 35233, USA; 5 Department of Family and Community Medicine, University of Alabama at Birmingham (UAB), Birmingham, AL 35205, USA

**Keywords:** cancer survivors, dyads, diet, exercise, self-efficacy, social support, barriers, internet

## Abstract

Mechanisms that explain behavior change within web-based lifestyle interventions are not well-studied. This secondary analysis explores whether the effects of the DUET web-based lifestyle intervention on diet, physical activity, and/or adiposity are mediated through changes in self-efficacy, social support, and perceived barriers (key constructs of social cognitive theory). Data on mediators, diet quality, caloric intake, moderate-to-vigorous physical activity (MVPA), weight, and waist circumference (WC) were analyzed from 112 cancer survivors and their partners enrolled in the DUET intervention. Mediation analyses were performed using Mplus to execute regression analyses and determine associations. Mediation analyses supported an effect of the intervention on caloric intake (−3.52, 95% CI [−8.08 to −0.84]), weight (−1.60, CI [−3.84 to −0.47]), and WC (−0.83, CI [−1.77 to −0.18]), interpreting these negative associations as intervention induced reductions in dietary barriers. Higher social support was significantly and positively associated with, but not a mediator for, improvements in self-reported and accelerometry-measured MVPA (b = 0.69, CI [0.19, 1.24]) and (b = 0.55, CI [0.15, 1.00]), respectively. Self-efficacy did not appear to mediate the intervention’s effects. Findings suggest that the effects of the DUET intervention on diet and adiposity stem from reducing perceived barriers to a healthful, low-calorie diet.

## 1. Introduction

More people are surviving cancer due to improvements in early cancer screening and advancements in reliable and effective treatments [1,2]. Hence, the number of cancer survivors continues to rise in the United States (U.S.) and now exceeds 18 million [1]. While improvements in cancer survivorship are encouraging, cancer survivors experience a multitude of challenges after diagnosis [3] and are at an increased risk for cancer recurrence, second primaries, functional decline, and other comorbidities such as cardiovascular disease, type 2 diabetes, and obesity [4,5,6,7,8]. Thus, healthcare costs for cancer survivors are substantial, and annual costs now exceed $183 billion per year (i.e., the estimated cost in 2015) [9]. 

As with the prevalence of cancer survivorship, obesity rates in the U.S., as well as among cancer survivors, are also growing [10,11]. In 2021, about 30% of cancer survivors aged 20 years or older were categorized as having obesity [11]. Large meta-analyses have reported that a weight gain of 5 kg/m^2^ is a significant predictor of cancer recurrence among breast and prostate cancer survivors [12,13]. Moreover, obesity also is known as a poor prognostic indicator for 15 different malignancies [13,14]. Thus, the American Cancer Society (ACS) and the World Cancer Research Fund-American Institute for Cancer Research (WCRF-AICR) have issued guidelines that support weight management and lifestyle factors that contribute to optimal energy balance for cancer survivors [15,16]. These guidelines include: (1) being at and maintaining a healthy weight (body mass index (BMI) of 18.5–24.9); (2) performing at least 150 min/week of moderate-to-vigorous physical activity (MVPA); and (3) eating a plant-based diet that is rich in vegetables, fruits, and whole grains, and low in red and processed meats, sugars, and ultra-processed foods. However, more than 80% of cancer survivors do not adhere to these diet and physical activity (PA) recommendations, and 69.2% fail to achieve and maintain a healthy weight [17,18]. 

Over the past decade, many multi-behavioral interventions have been implemented among cancer survivors to improve diet and PA behaviors and target weight loss [19,20,21,22]. However, the most effective behavioral interventions are the ones that employ theoretical frameworks and use behavioral theories to guide sustainable dietary and PA practices [23]. While interventionists have used a variety of behavioral theories (i.e., transtheoretical model, the theory of planned behavior, and cognitive behavioral therapy) to promote behavior change among cancer survivors, the Social Cognitive Theory (SCT) is one of the most robust [19,20,24]. SCT is based on the foundation of dynamic and reciprocal interaction that emphasizes the importance of concepts such as self-efficacy, e.g., one’s confidence in their ability to perform behaviors, as well as social and environmental factors (i.e., social support from family and friends and overcoming perceived barriers) to adopt healthy behaviors [25]. Thus, these SCT constructs have been evaluated as working mechanisms through which traditionally delivered (e.g., face-to-face, telephone counseling, and mailed print) multi-behavioral interventions promote diet and PA behaviors among cancer survivors [26,27]. 

For instance, the FRESH START trial, a personally-tailored mailed print diet and PA intervention conducted among 543 cancer survivors, was informed by the SCT and reported improvements in self-efficacy for diet as a key mediator for long-term increases in vegetable and fruit (V&F) consumption and decreases in fat intake at 2-year follow-up [26]. Additionally, the BEAT Cancer program, also guided by the SCT, implemented an in-person 12-week individual and group PA intervention to gradually increase PA among 41 breast cancer survivors [27]. The study reported that the effect of the BEAT intervention on PA at 3-month follow-up was mediated through a reduction in barrier interference. Likewise, studies have identified the importance of incorporating social networks within interventions to improve diet and PA behaviors, given the role that friends and family play during cancer diagnosis and treatment [28,29]. Hence, these findings suggest that traditionally delivered interventions among cancer survivors can encourage positive dietary and PA practices through enhancing self-efficacy and minimizing barriers, as well as incorporating strategies that bolster social support. However, current knowledge regarding mediation is largely limited to interventions that are delivered via in-person or telephone counseling and mailed materials programming. 

The interest of cancer survivors is shifting towards online programs to gain health information [30]. This trend could partially be due to the expansion of web-based modalities during the COVID-19 pandemic [31]. Hence, investigators are implementing diet and PA interventions using more scalable, personalized, cost-effective web-based platforms [32,33,34,35]. The Daughters, dUdes, mothers, and othErs Together (DUET) trial was a 6-month web-based lifestyle intervention delivered among 112 cancer survivors and their partners (56 dyads) with overweight or obesity to promote weight loss [36,37]. DUET was adapted from a tailored mailed print intervention, Daughters and Mothers Against Breast Cancer (DAMES), which was grounded on SCT [38]. However, DUET incorporated newer technologies, such as an interactive website, fitness trackers, and digital scales, to promote healthy diet and PA behaviors, and weight loss. The website supported a personalized approach to healthy eating and exercising by incorporating interactive e-learning videos, tracking and self-monitoring, role-modeling strategies for internal motivation, and tailored social support through a buddy system approach. The primary outcomes revealed that dyads randomized to the DUET intervention reported significant reductions in body weight (−2.8 kg) and caloric intake (−164.1 kcals) compared to dyads in the wait-list control arm, −1.1 kg and −99.2 kcals, respectively [37]. However, while significant within-arm improvements in diet quality (+3.3 Healthy Eating Index (HEI)), MVPA (+13.5 min), and waist circumference (WC) (−3.3 cm) were detected among dyads in the intervention arm, no statistically significant differences were observed between arms [37]. 

To date, very few studies have identified the SCT-mediating processes through which multi-behavior web-based interventions change health behavior among cancer survivors. Therefore, evaluating constructs that have been shown to be effective in improving diet and PA behaviors in previous, more traditionally delivered interventions is key to understanding behavior change using this newer technology. To address this gap, the current study explores whether the effects of the DUET web-based lifestyle intervention on diet, MVPA, and/or adiposity are mediated through SCT constructs (self-efficacy, social support, and perceived barriers to diet and exercise). We hypothesize that the previously reported [37] improvements in diet, MVPA, and/or adiposity among cancer survivor-partner dyads randomized to the DUET web-based lifestyle intervention (vs. control arm) are mediated through improvements in self-efficacy and social support and reductions in perceived barriers. 

## 2. Materials and Methods

### 2.1. Study Design and Participants

A secondary analysis was performed using baseline and 6-month follow-up data from the DUET intervention, a 2-arm, single-blinded, randomized controlled trial (RCT) in which 112 cancer survivors and their partners (56 dyads) were assigned to either a 6-month web-based diet and exercise, weight loss intervention or a wait-list control. The study was approved by the University of Alabama at Birmingham (UAB) Institutional Review Board (IRB# 300003882) and registered with ClinicalTrials.gov (NCT04132219). All participants in the study provided written informed consent. Data collection started in October 2020 and ended in December 2021. The methods and primary outcomes of the DUET trial are published in detail elsewhere [36,37]. 

Cancer survivors were ascertained through a combination of cancer registries, self-referral networks, and previously established lists of interested cancer survivors who were diagnosed and completed treatment for obesity-related cancers that have a 5-year survival rate of ≥70% (i.e., localized renal cancer and loco-regional ovarian, colorectal, prostatic, endometrial, and female breast cancers). These individuals were telephoned and screened by the study staff. The eligibility criteria for the DUET study were as follows: (1) BMI > 25 kg/m^2^; (2) V&F intake < 2.5 cups/day; (3) MVPA < 150 min/week; and (4) regular Internet use and mobile phone ownership. Eligible cancer survivors were asked to identify a local partner who lived within a 10-minute driving distance and with whom they interacted at least biweekly; similar eligibility criteria were used to screen partners (exception: cancer diagnosis). 

### 2.2. DUET Intervention and Mediators

The DUET intervention utilized newer and personalized technologies, i.e., interactive website, Inspire Fitbit® fitness trackers, and Aria 2 digital scales (Fitbit, Inc. San Francisco, CA, USA) to improve diet and PA behaviors among cancer survivors and their partners. Details on the intervention content are described in a separate publication [36]. In brief, DUET was theoretically grounded by SCT and incorporated several behavioral change techniques (BCTs) to target key constructs of SCT, e.g., self-efficacy, social support, and perceived barriers [39]. The DUET intervention aimed to reduce perceived barriers and improve social support and self-efficacy for diet and PA by providing access to a self-guided website (https://duet4health.org, accessed on 4 September 2023) that offered a serialized program of 24 interactive educational e-learning sessions that were prompted by SMS text messages, as supported by the provision of the fitness tracker and digital scale. A key feature of the DUET website was the weekly self-guided e-learning sessions, which focused on providing instructions on making healthful dietary and PA choices, incremental goal setting, guidance for overcoming common barriers to a healthful diet and regular exercise, and practical strategies to guide long-term healthy behaviors. The website provided personalized progress reports to track diet and exercise behaviors and weight status. The website was tailored to both the survivor (who received additional information on how to overcome common treatment-related barriers to achieve goals) and the partner and also encouraged helpful strategies on how to request and provide instrumental, informational, appraisal, and emotional support [40]. Additionally, the “Healthy Weight”, “Healthy Eating”, “Exercise”, and “Tools” features provided key information. A total of three SMS text messages were sent each week, which focused on push, support, and action messages to engage participants with the website. The Inspire Fitbit^®^ fitness tracker and Aria 2 digital scale provided guidance on tracking exercise activities and weight to enhance self-monitoring and internal motivation. Details on how the DUET intervention targeted self-efficacy, social support, and perceived barriers to diet and exercise and specified BCTs using the CALO-RE taxonomy [39] are described in Table S1.

### 2.3. Measures

#### 2.3.1. Dependent Variables (Y) 

Two-day dietary recalls were conducted at baseline and 6-months via telephone by a registered dietitian on one weekday and one weekend day using the National Cancer Institute (NCI)-developed Automated Self-Administered 24-h (ASA24) dietary recall assessment web-based tool (https://epi.grants.cancer.gov/asa24, accessed on 4 September 2023). Diet quality and average caloric intake were measured using the HEI-2015 [41]. 

To measure MVPA, both objective and subjective data were collected at baseline and 6 months. ActiGraph accelerometers were programmed to capture objective PA over a 7-day period. The data from accelerometers were downloaded and processed with the ActiLife software (version 6.13 Fort Walton, FL, USA), using procedures supplied by the manufacturer and methods similar to those reported previously to obtain MVPA [42]. For subjective measures, self-reported PA was measured using the Godin Leisure Time Exercise Questionnaire (GLTEQ) due to its strong reliability and validity among cancer survivors and ease of use [43].

Adiposity was assessed remotely by capturing data on both body weight and WC at baseline and 6 months. The study staff scheduled a remote assessment via Zoom^®^ (San Jose, CA, USA) with both dyad members together. Remote assessment materials, instructions, and a video (https://youtu.be/G8p6g_VDzhw, accessed on 4 September 2023) were provided to participants in advance of the assessment. To measure weight, participants were asked to use a bathroom scale. A digital scale was provided with the assessment materials for participants who did not own a scale. During the Zoom call, a trained staff member advised participants to wear light clothing and remove their shoes during weighing. The scale was “zeroed-out” prior to weighing, and weight was measured twice and averaged for analysis. WC was measured twice on each dyad member using two sets of unmarked ribbons, as per the methodology proposed by Freudenheim and Darrow [44]. The two sets of ribbons were returned to the study office, where they were measured by trained staff members. An average was used for the analysis. Details on the assessment procedures, reliability, and validity of remote assessments are discussed in a separate publication [45]. 

#### 2.3.2. Mediators (M)

Self-efficacy for diet was measured using a 20-item instrument (internal consistencies = 0.70–0.88) on 5 subscales (i.e., negative emotions, availability, social pressure, physical discomfort, and positive activities) for high-calorie foods by Clark et al. [46]. Confidence to resist eating high-calorie foods was assessed using a Likert-scaled percentage (0% = not confident to 100% = extremely confident). Mean percent scores were used for the analysis, with higher scores indicating greater self-efficacy for resisting high-calorie foods. Self-efficacy for exercise was measured using the 8-item Lifestyle Efficacy scale (internal consistency = 0.95) for exercise by McAuley et al. [47]. The scale asked participants to report their confidence (0% = not confident to 100% = extremely confident) under specific conditions: (1) When I lack the discipline to exercise; (2) When exercise is not a priority; (3) When weather is bad; (4) When I am tired; (5) When I am not interested in exercising; (6) When I lack time; (7) When I do not enjoy exercising; and (8) When I do not have someone to encourage me to exercise. Mean percent scores were used for analysis, with higher scores indicating greater self-efficacy for exercising. 

Social support for diet was measured using a validated (r = 0.55–0.86 and internal consistencies = 0.61–0.91) 4-item instrument on 3 subscales (i.e., participation, involvement, and encouragement) by Sallis et al. [48] and assessed using a 5-point Likert-scale (1 = Never to 5 = Every day). Participants were asked: (1) How often have your family or friends listened to your concerns about eating a healthy diet?; (2) How often have your family or friends assisted you in eating a healthy diet?; (3) How often have your family or friends agreed with your decisions to eat a healthy diet?; and (4) How often have your family or friends encouraged choices favorable to eating a healthy diet? Mean scores were used for the analysis, with higher scores indicating greater support for diet. Social support for exercise was measured using a similar methodology. 

The frequency of encountering barriers to following a low-calorie, healthful diet was assessed using a 10-item questionnaire with anchors ranging from 1 = Never to 5 = Every day [49,50]. The percentage of times the participant reported “often” or “everyday” out of 10 questions were summed, with higher scores indicating higher perceived barriers to adhering to a low-calorie diet. Perceived barriers to exercise were measured using a 21-item questionnaire with identical anchors [49,51]. The percentage of times the participant reported “often” or “everyday” out of 21 questions were summed, with higher scores indicating higher perceived barriers to exercising.

### 2.4. Simple Mediation Model

**Figure 1:**
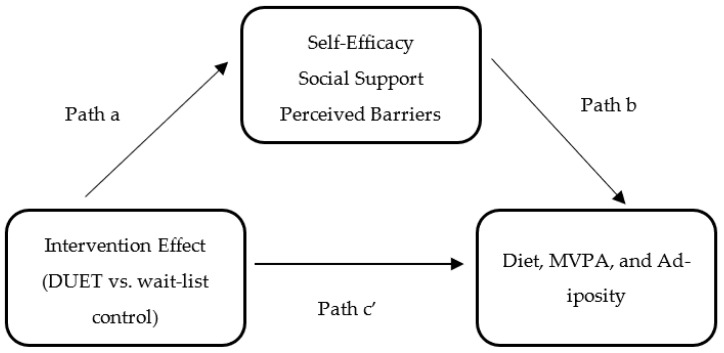
Simple mediation model for the hypothesized mediators: Evaluating the effects of the DUET web-based lifestyle intervention on diet, moderate-to-vigorous physical activity (MVPA), and adiposity through self-efficacy, social support, and perceived barriers.

To examine whether the hypothesized mediators had a mediation effect, the following three conditions, as depicted in Figure 1, were assessed: (1) the effect of the DUET intervention on self-efficacy, social support, and perceived barriers (path a); (2) the effect of self-efficacy, social support, and perceived barriers on diet, MVPA, and adiposity while controlling for the intervention effect (path b); and (3) the direct effect (path c’) of the DUET intervention on diet, MVPA, and adiposity while controlling for the mediators. The indirect effect (ab) of the DUET intervention on diet, MVPA, and adiposity through self-efficacy, social support, and perceived barriers was calculated using the product of the standard estimates approach (path a*path b). The overall effect of the DUET intervention on diet, MVPA, and adiposity was classified as the total effect, which is also equal to the sum of the direct and indirect effects.

### 2.5. Statistical Analysis

All statistical analyses were performed using structural equation modeling software, Mplus (version 8, Muthen & Muthen, Los Angeles, CA, USA) [52]. Simple mediation analyses on 112 cancer survivors and partners were performed to identify whether the effect of the DUET web-based lifestyle intervention on diet, MVPA, and adiposity was mediated through self-efficacy, social support, or perceived barriers. To estimate the power within the sample, simulation results were used, as presented by Fritz and MacKinnon [53]. With 112 participants and assuming randomization to the intervention group is associated with a 0.26 standard deviation change in the mediating variable, and a one standard deviation change in the mediating variable, in turn, is associated with a 0.59 standard deviation change in the dependent variable, the study had a power of 0.80 to detect indirect effects. Intervention assignment (X) was indicated by a dichotomous variable (coded as DUET intervention = 1 vs. wait-list control = 0). Dependent variables (Y) (i.e., diet quality, calories, MVPA, weight, and WC) and mediators (M) (i.e., self-efficacy, social support, and perceived barriers) were measured on a continuous scale. The normality of errors for dependent and mediating variables was evaluated using the Kolmogorov–Smirnov tests and quantile distributions, which indicated non-normal distributions for MVPA, weight, social support, and perceived barriers due to extreme values. To test for homoscedasticity, residuals were plotted against predicted values to identify whether the variance of regression errors was similar across the estimated value. The linearity of the association between mediating and dependent variables was assessed using scatter plots, which suggested non-linear associations in MVPA models. Hence, a hyperbolic sine transformation for MVPA was used to generate more linear associations, as well as reduce skewness and improve the consistency of variances across levels of the independent variables [54]. Additionally, given the dyadic nature of the DUET intervention, cancer survivors and their partners could potentially influence one another, thus violating the assumption of independence. To account for non-independence, clustering syntax was used to control multilevel data. Due to non-normality and heteroscedastic errors in the data, standard estimates were evaluated using maximum likelihood (ML) parameter estimates plus bootstrapping at 500 bootstrap resamples, a non-parametric resampling approach. Model parameters were estimated using full information maximum likelihood (FIML) estimation, which uses all available data for estimation and compares favorably to multiple imputations in handling missing data [55]. The effects represent the change in self-efficacy, social support, and perceived barriers from baseline to 6 months as potential mediators of the intervention’s effect on diet quality, MVPA, and adiposity at 6 months. Thus, based on the disjunctive cause criterion approach [56], baseline values of the dependent and mediating variables were used as covariates in each model in addition to age, sex, and race. The data reports unstandardized parameter estimates, standard errors (SE), the 95% confidence interval (CI), and r-squared values (to detect goodness of fit) for each model. An alpha level of 0.05 was used to detect significant associations, and 95% CI’s that did not include zero provided evidence for significance from bootstrap results for paths a, b, c’, and indirect and total effects.

## 3. Results

Sample characteristics and main outcomes of the DUET study were published previously [37]. In brief, the mean age of the study sample was 58 years, with the majority being female (76.8%), non-Hispanic White (62.5%), and residents of urban areas (92%). Further, roughly half were fully employed (55.4%) and college graduates (53.5%); 42.8% reported annual incomes > $50,000. Most cancer survivors had diagnoses of breast cancer (80.3%), with stage I cancer (41.1%) being the most prevalent; the average time elapsed since diagnosis was 5.6 years. 

**Figure 2:**
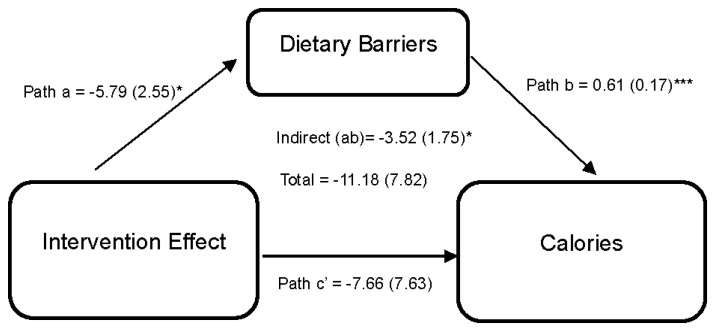
Model represents the effects of the DUET intervention on calories through perceived dietary barriers. All models showed mediation through perceived dietary barriers at 6 months, controlling for baseline dependent and mediator variables as well as age, race, and sex. The unstandardized parameter estimates followed by standard errors in parentheses are reported for each path and indirect and total effects. * *p* < 0.05 and *** *p* < 0.001.

Statistically significant differences from baseline to 6 months in diet and exercise perceived barriers were observed among dyads in the intervention arm. However, no statistically significant differences were observed between arms. While improvements in diet and exercise, social support, and self-efficacy were reported in the intervention compared to the wait-list control during the 6-month period, it did not achieve statistical significance. Figure 2 shows the effect of the DUET intervention on caloric intake through perceived dietary barriers. Results indicate that the DUET intervention was associated with reductions in perceived dietary barriers (path a = −5.79 (2.55), 95% CI [−11.48, −1.26]). In turn, individuals who reported higher perceived dietary barriers reported higher caloric intake (path b = 0.61 (0.17), 95% CI [0.28,0.94]). Thus, part of the overall effect of the DUET intervention on caloric intake was through reducing perceived dietary barriers, as indicated by a significant indirect effect (ab = −3.52 (1.75), 95% CI [−8.08, −0.84]). 

**Figure 3:**
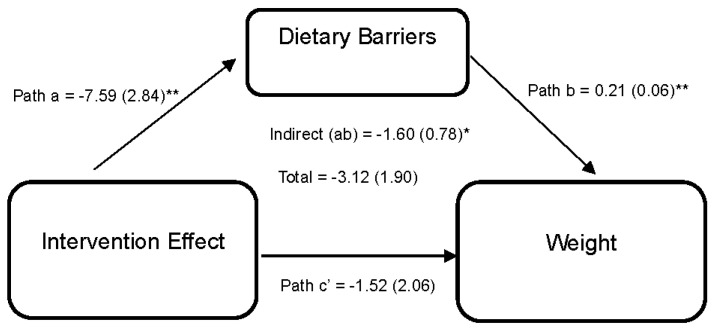
Model represents the effects of the DUET intervention on weight through perceived dietary barriers. All models show mediation through perceived dietary barriers at 6 months, controlling for baseline dependent and mediator variables as well as age, race, and sex. The unstandardized parameter estimates followed by standard errors in parentheses are reported for each path and indirect and total effects. * *p* < 0.05 and ** *p* < 0.01.

Figure 3 illustrates the effect of the DUET intervention on weight through perceived dietary barriers. Results reveal that the DUET intervention was associated with reductions in perceived dietary barriers (path a = −7.59 (2.84), 95% CI [−13.68, −2.18]). In turn, individuals who reported higher perceived dietary barriers reported higher weight (path b = 0.21 (0.06), 95% CI [0.08,0.32]). Thus, part of the overall effect of the DUET intervention on weight was by reducing perceived dietary barriers, as indicated by a significant indirect effect (ab = −1.60 (0.78), 95% CI [−3.84, −0.47]).

**Figure 4:**
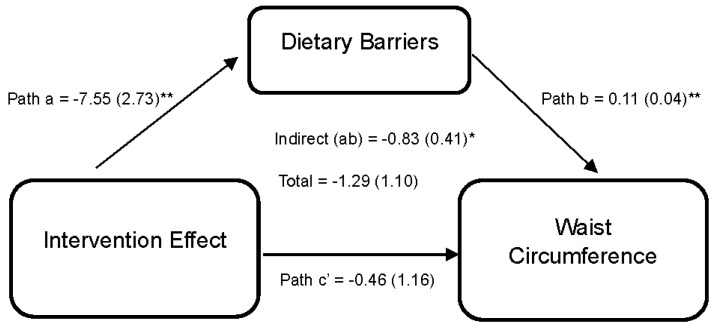
Model represents the effects of the DUET intervention on waist circumference through perceived dietary barriers. All models show mediation through perceived dietary barriers at 6 months, controlling for baseline dependent and mediator variables as well as age, race, and sex. The unstandardized parameter estimates followed by standard errors in parentheses are reported for each path and indirect and total effects. * *p* < 0.05 and ** *p* < 0.01.

Lastly, Figure 4 reports the effect of the DUET intervention on WC through perceived dietary barriers. Results indicate that the DUET intervention was associated with reductions in perceived dietary barriers (path a = −7.55 (2.73), 95% CI [−13.68, −2.72]). In turn, individuals who reported higher perceived dietary barriers reported higher WC, on average (path b = 0.11 (0.04), 95% CI [0.01,0.16]). Thus, part of the overall effect of the DUET intervention on WC was through reducing perceived dietary barriers, as indicated by a significant indirect effect (ab = −0.83 (0.41), 95% CI [−1.77, −0.18]).

Overall, significant indirect effects support the effect of the DUET intervention on caloric intake, weight, and WC through reductions in perceived dietary barriers. However, significant mediating effects were not detected for diet quality or MVPA. Details on direct and total effects, as well as unstandardized parameter estimates, SE, 95% CI, and r^2^ for each model, are shown in Table 1.

Table 2 reports the effects of the DUET intervention on diet, MVPA, and adiposity through social support. Higher exercise social support was significantly associated with improvements in self-reported and accelerometer-measured MVPA at 6 months (path b = 0.69 (0.27), 95% CI [0.19, 1.24]) and (path b = 0.55 (0.22), 95% CI [0.15, 1.00]), respectively. However, social support was not a significant mediator for the DUET intervention due to the minimal effect of the DUET intervention on social support (non-significant effects for path a in each model).

Likewise, Table 3 reports the effects of the DUET intervention on diet, MVPA, and adiposity through self-efficacy. Self-efficacy, a key behavioral construct of SCT, also did not appear to mediate the effects of the DUET intervention on diet, MVPA, or adiposity due to the minimal effect of the DUET intervention on self-efficacy (non-significant effects for path a in each model).

## 4. Discussion

Scalable and effective diet and PA interventions that promote weight management among cancer survivors, as well as their family and friends, are needed [28]. DUET is one of the first web-based studies to achieve significant weight loss in both cancer survivors and partners and was also found to have favorable effects on diet and PA behaviors [37]. Like many effective interventions, it was designed using SCT as a theoretical foundation, but it was unique in that it was delivered totally through a web-based platform and used newer technologies such as an interactive website, fitness trackers, and digital scales. Hence, we had an opportunity to explore whether the SCT constructs that have been shown to mediate other traditionally delivered interventions operated in the same way within DUET. To our knowledge, this current study is one of the first to perform mediation analysis on a web-based, multi-behavior clinical trial of diet and PA and is a pioneering effort in this new age of web-based interventions.

Surprisingly, self-efficacy—one of the more robust constructs of SCT—was not found to mediate DUET’s effect on diet, MVPA, and adiposity. These results differ remarkably from larger, traditionally delivered interventions among cancer survivors [26,57]. In the personally tailored, mailed print FRESH START intervention conducted among 543 breast and prostate cancer survivors, improvements in diet self-efficacy explained long-term changes in diet at a 2-year follow-up [26]. Similarly, more recent work by Kindred et al. reported that the adoption of PA was explained through significant improvements in exercise self-efficacy in a telephone intervention for breast cancer survivors [57]. However, DUET is not the only study that did not achieve significant improvements in self-efficacy and failed to show mediation; other investigators have found similar results [58,59]. A partial explanation for our inability to affect self-efficacy within the DUET intervention could be due to challenges in achieving strong engagement with the website platform. A previous finding by Leslie et al. [60] and a more recent report by Li et al. [61] indicated that participants enrolled in web-based programs show decreases in website engagement over time. Hence, incorporating BCTs that enhance motivation at specific intervals throughout the intervention period may be helpful in improving engagement. Moreover, DUET was delivered during the COVID-19 pandemic, an unprecedented time when loneliness, depression, anxiety, and stress were worsened among the U.S. population, as well as cancer survivors [62,63]. These psychosocial challenges, among others, may have hindered motivation and engagement with the dyad-based intervention.

Social support also was not found to mediate DUET’s effects on diet, MVPA, and adiposity. Our findings are similar to other website-based lifestyle interventions conducted among U.S. adults, such as the Guide-to-Health [64] and e-coachER [65] trials. The Guide-to-Health trial utilized a website and church networks to provide social support. Yet, significant changes in dietary social support were not observed. Similarly, the e-coachER trial failed to detect improvements in support frequency, which did not mediate the intervention’s effect on MVPA further. The challenge in targeting social support in this current study and others may stem from the quality and availability of social networks, factors that may influence support received during the intervention [28,66]. Our results and these others contradict a recent large group-based Texercise Select trial [67], in which participants in the intervention arm reported significant improvements in social support compared to the control arm, and social support was found to be a significant mediator of the intervention’s effect on diet. However, unlike our study that measured dietary, social support from family and friends, Texercise Select measured diet-specific social support (i.e., support for planning dietary goals, keeping dietary goals, and reducing barriers to healthy eating) and was much larger in size (n = 386) compared to this current study (n = 112). Hence, including different measures of social support (i.e., instrumental, emotional, and diet and exercise specific) and evaluating the quality and availability of social networks prior to enrollment should be considered when targeting social support within lifestyle interventions that are delivered using web-based platforms.

Although self-efficacy and social support were not found to mediate DUET’s effect on diet, PA, and adiposity, our results found a significant mediating effect on caloric intake, weight, and WC through reductions in dietary barriers. These findings concur with the results of mediation analyses from many traditionally delivered lifestyle interventions [68]. A systematic review by Teixeira et al. [68] of 35 lifestyle interventions delivered among adults identified self-efficacy barriers and perceived barriers as mediators for dietary intake and weight control. It should be noted that the majority of interventions in this review were implemented using face-to-face methods. Of note, a recent mHealth lifestyle intervention also reported that reductions in dietary barriers significantly predicted improvements in dietary intake; [69] however, due to the limited sample size, the team did not conduct a formal mediation analysis. Thus, our results corroborate the findings of most studies that support the role of barrier reduction in improving dietary behaviors. However, our findings contrast with those of the North Carolina Strategies for Improving Diet, Exercise, and Screening (NC STRIDES) study [70] conducted among colorectal cancer survivors and individuals without cancer, as well as a culturally tailored, family-based intervention conducted among Latina mothers [71], that did not find evidence of such mediation. DUET’s more powerful effect on dietary barriers may have stemmed from its relatively longer duration (6 months) and its weekly interactive and self-paced e-learning sessions that were geared toward eliciting responses that promoted knowledge, engagement, and progression throughout the intervention. Moreover, the DUET website also included online resources and tools that participants could utilize to overcome barriers to food procurement and preparation to make healthy eating easier and more attainable.

In contrast, our study results did not show that exercise barriers were a significant mediator of DUET’s effect on MVPA. Similar to dietary barriers, the DUET intervention focused on overcoming exercise-related barriers through weekly, self-paced website e-learning sessions that provided strategies to overcome common barriers (e.g., lack of time or energy), as well as barriers that are more specific and treatment-related (e.g., pain or urinary incontinence during exercise). However, our study did not show evidence of mediation through a reduction in perceived barriers to exercise and, therefore, is similar to an app-based physical activity program conducted among adults, as well as other studies utilizing web-based platforms [72,73,74]. In contrast, a face-to-face counseling intervention delivered among breast cancer survivors, the BEAT trial [27], showed significant improvements in MVPA through reductions in exercise barrier interference. This disagreement in results between web-based versus traditionally delivered interventions could be explained partially by differences in the mode of delivery. For instance, while cancer survivors may have retained information on the benefits of performing PA from the DUET e-learning sessions, certain exercise barriers such as lack of equipment, access to skilled instructors, financial and time constraints, and availability of gym facilities are more difficult to achieve within web-based platforms as compared to in-person programs.

There are several strengths of this current study. DUET included a heterogeneous sample of cancer survivors and their partners in age (23–81 years) and broad reach across the U.S. (Alabama, Illinois, Mississippi, North Carolina, and Tennessee). Additionally, DUET enrolled dyads with diverse family relationships and social networks (i.e., spouse, child, siblings, and friends) as compared to other dyadic interventions delivered among cancer survivors and their spouses or children [38,75]. To our knowledge, this study is among the few to examine individual, social, and environmental constructs of SCT theory to provide an understanding of how the DUET web-based lifestyle intervention improved diet, MVPA, and adiposity among cancer survivors and their partners. Moreover, our study utilized validated multi-item scales for self-efficacy and social support and included more than one measure for diet, MVPA, and adiposity. However, like all studies, there were limitations, with the primary shortcoming being the modest sample size to conduct mediation analysis. Fritz and MacKinnon suggest a need for a median sample size of 143–150 to detect indirect effects [53]; hence, our inability to detect mediating effects for self-efficacy and social support may be due to a lack of power. Additionally, while this study used reliable multi-item social support and self-efficacy questionnaires that capture greater variability, they may underestimate effect sizes due to an inability to target specific components of self-efficacy and social support utilized within web programs. Furthermore, although the bank of items for perceived dietary and exercise barriers was adapted and has been used in previous studies of cancer survivors [49,51], they were not validated in this specific population of survivors and partners. Other limitations were a lack of establishing temporal precedence and inadequate representation of males, rural survivors, and survivors of cancer types other than breast cancer. However, it must be remembered that all secondary analyses, by their very nature, are hypothesis-generating and not hypothesis testing—thus calling for additional investigation.

## 5. Conclusions

The use of web-based interventions is increasing in all populations, including cancer survivors. Online programs increase scalability and can maximize standardization and fidelity to health behavior theory to promote lifestyle change. However, there is a need to understand theoretical constructs that lead to behavior change. The present findings suggest that the effect of the DUET web-based intervention on diet and adiposity stems from reducing perceived barriers to a healthy, low-calorie diet. However, the intervention effects on self-efficacy and social support, along with our small sample size, likely contributed to null findings related to the lack of mediation by these constructs. Therefore, in applying these findings to the field of health education, particularly the growing field of eHealth, the ungirding of behavioral theory (whether it be social cognitive theory or another) and the systematic application of BCTs appears key in understanding and promoting behavior change. Health educators who deliver these interventions or those who procure eHealth programs for patient populations and other end-users need to familiarize themselves with the “active ingredients” that lead to desired outcomes, such as weight loss or improved health. In the case of DUET, we found that by reducing perceived barriers to a low-calorie diet, we may have been able to achieve the reductions in calorie intake and subsequent weight loss that were sought. Findings from this study should be replicated in future website-based interventions, implemented on a larger scale, to understand the “active ingredients” behind successful web-based lifestyle interventions.

## Figures and Tables

**Table 1:** Simple mediation analysis: Assessing indirect effects of the DUET intervention on outcome measures through dietary and exercise barriers.

		Consequent									
Antecedent			Dietary Barriers (M)				Diet Quality (Y)			Indirect and Total Effects (SE, Standard Errors, [95% CI])	
			Coeff.	SE	95% CI		Coeff.	SE	95% CI	ab	Total
DUET vs. WLC (X)		a	−6.49	2.60	−12.33, −1.93	c’	3.38	3.03	−2.14, 9.32	0.62 (0.57) [−0.15, 2.15]	4.00 (2.95) [−1.33, 9.71]
Dietary Barriers (M)			-	-		b	−0.10	0.09	−0.26, 0.06		
Constant		i_M_^1^	10.40	8.43	−4.00, 29.53	i_Y_^2^	38.48	10.54	15.99, 57.55		
			R^2^ = 0.240, *p* = 0.003				R^2^ = 0.111, *p* = 0.222				
			Dietary Barriers (M)				Calories (Y)			−3.52 (1.75) [−8.08, −0.84]	−11.18 (7.82) [−27.10, 2.91]
DUET vs. WLC (X)		a	−5.79	2.55	−11.48, −1.26	c’	−7.66	7.63	−23.38, 5.51		
Dietary Barriers (M)			-	-	-	b	0.61	0.17	0.28, 0.94		
Constant		i_M_	3.63	8.51	−13.66, 20.77	i_Y_	68.94	22.71	17.53, 112.05		
			R^2^ = 0.251, *p* = 0.001				R^2^ = 0.497, *p* =< 0.001				
			Exercise Barriers (M)				Self-report MVPA (Y)			0.16 (0.12) [−0.01, 0.51]	0.86 (0.42) [0.15, 1.75]
DUET vs. WLC (X)		a	−6.13	3.48	−12.47, 1.11	c’	0.70	0.43	−0.06, 1.55		
Exercise Barriers (M)			-	-	-	b	−0.03	0.01	−0.06, −0.00		
Constant		i_M_	15.11	8.86	−3.66, 32.96	i_Y_	2.56	1.34	−0.16, 4.97		
			R^2^ = 0.271, *p* = 0.007				R^2^ = 0.312, *p* = 0.001				
			Exercise Barriers (M)				Accelerometer MVPA (Y)				
DUET vs. WLC (X)		a	−5.92	3.89	−13.42, 2.90	c’	0.32	0.44	−0.61, 1.08	0.03 (0.08) [−0.10, 0.24]	0.35 (0.42) [−0.58, 1.10]
Exercise Barriers (M)			-	-	-	b	−0.00	0.01	−0.03, 0.02		
Constant		i_M_	18.78	12.18	−1.12, 45.54	i_Y_	2.31	1.35	−0.71, 4.87		
			R^2^ = 0.270, *p* = 0.011				R^2^ = 0.329, *p* = 0.002				
			Dietary Barriers (M)				Weight (Y)				
DUET vs. WLC (X)		a	−7.59	2.84	−13.68, −2.18	c’	−1.52	2.06	−5.66, 2.24	−1.60 (0.78) [−3.84, −0.47]	−3.12 (1.90) [−7.08, 0.38]
Dietary Barriers (M)			-	-	-	b	0.21	0.06	0.08, 0.32		
Constant		i_M_	33.18	14.53	4.78, 62.23	i_Y_	6.35	8.27	−10.79, 21.12		
			R^2^ = 0.276, *p* = 0.001				R^2^ = 0.951, *p* =< 0.001				
			Dietary Barriers (M)				Waist Circumference (Y)				
DUET vs. WLC (X)		a	−7.55	2.73	−13.68, −2.72	c’	−0.46	1.16	−2.67, 1.82	−0.83 (0.41) [−1.77, −0.18]	−1.29 (1.10) [−3.51, 0.87]
Dietary Barriers (M)			-	-	-	b	0.11	0.04	0.01, 0.16		
Constant		i_M_	43.76	15.41	15.25, 74.48	i_Y_	6.18	7.21	−8.53, 19.21		
			R^2^ = 0.289, *p* =< 0.001				R^2^ = 0.862, *p* =< 0.001				

^1^ i_M_ = intercept; ^2^ i_Y_ = intercept.

**Table 2:** Simple mediation analysis: Assessing indirect effects of the DUET intervention on outcome measures through dietary and exercise social support.

		Consequent									
Antecedent			Dietary Social Support (M)				Diet Quality (Y)			Indirect and Total Effects (SE, Standard Errors, [95% CI])	
			Coeff.	SE	95% CI		Coeff.	SE	95% CI	ab	Total
DUET vs. WLC (X)		a	0.18	0.12	−0.08, 0.40	c’	3.60	2.79	−1.51, 9.39	0.49 (0.54) [−0.21, 2.02]	4.09 (2.77) [−0.89, 9.81]
Dietary Social Support (M)			-	-	-	b	2.82	1.87	−1.07, 6.08		
Constant		i_M_^1^	1.88	0.42	1.09, 2.7	i_Y_^2^	26.30	12.01	3.10, 48.73		
			R^2^ = 0.323, *p* =< 0.001				R^2^ =0.133, *p* = 0.176				
			Dietary Social Support (M)				Calories (Y)			1.09 (1.15) [−0.17, 5.58]	−11.53 (7.67) [−26.95, 2.07]
DUET vs. WLC (X)		a	0.18	0.12	−0.09, 0.41	c’	−12.62	7.70	−28.33, 0.98		
Dietary Social Support (M)			-	-	-	B	6.20	4.37	−1.55, 17.50		
Constant		i_M_	2.22	0.53	1.29, 3.25	i_Y_	71.84	28.69	13.99, 125.16		
			R^2^ = 0.310, *p* =< 0.001				R^2^ = 0.476, *p* =< 0.001				
			Exercise Social Support (M)				Self-report MVPA (Y)			0.14 (0.14) [−0.04, 0.52]	0.84 (0.43) [0.10, 1.72]
DUET vs. WLC (X)		a	0.19	0.15	−0.08, 0.47	c’	0.70	0.42	−0.052, 1.55		
Exercise Social Support (M)			-	-	-	B	0.69	0.27	0.19, 1.24		
Constant		i_M_	1.44	0.53	0.43, 2.46	i_Y_	−0.92	1.32	−3.92, 1.23		
			R^2^ = 0.267, *p* =< 0.001				R^2^ = 0.283, *p* = 0.002				
			Exercise Social Support (M)				Accelerometer MVPA (Y)				
DUET vs. WLC (X)		a	0.20	0.17	−0.09, 0.57	c’	0.27	0.42	−0.60, 1.03	0.11 (0.11) [−0.04, 0.39]	0.38 (0.42) [−0.56, 1.11]
Exercise Social Support (M)			-	-	-	B	0.55	0.22	0.15, 1.00		
Constant		i_M_	1.72	0.74	0.22, 3.19	i_Y_	1.50	1.42	−1.41, 4.80		
			R^2^ = 0.273, *p* = 0.002				R^2^ = 0.356, *p* =< 0.001				
			Dietary Social Support (M)				Weight (Y)				
DUET vs. WLC (X)		a	0.18	0.12	−0.09, 0.41	c’	−3.13	1.90	−6.61, 0.51	−0.15 (0.36) [−1.10, 0.39]	−3.28 (1.90) [−7.03, 0.44]
Dietary Social Support (M)			-	-	-	B	−0.81	1.50	−3.31, 2.83		
Constant		i_M_	2.04	0.77	0.49, 3.36	i_Y_	15.24	10.24	−6.54, 32.86		
			R^2^ = 0.311, *p* =< 0.001				R^2^ = 0.945, *p* =< 0.001				
			Dietary Social Support (M)				Waist Circumference (Y)				
DUET vs. WLC (X)		a	0.19	0.13	−0.09, 0.42	c’	−1.09	1.08	−3.12, 1.05	−0.09 (0.24) [−0.89, 0.17]	−1.19 (1.09) [−3.39, 0.93]
Dietary Social Support (M)			-	-	-	B	−0.51	0.91	−2.23, 1.30		
Constant		i_M_	1.85	0.80	0.43, 3.60	i_Y_	9.51	9.26	−8.76, 26.48		
			R^2^ = 0.313, *p* =< 0.001				R^2^ = 0.850, *p* =< 0.001				

^1^ i_M_ = intercept; ^2^ i_Y_ = intercept.

**Table 3:** Simple mediation analysis: Assessing indirect effects of the DUET intervention on outcome measures through dietary and exercise self-efficacy.

		Consequent									
Antecedent			Dietary Self-Efficacy (M)				Diet Quality (Y)			Indirect and Total Effects (SE, Standard Errors, [95% CI])	
			Coeff.	SE	95% CI		Coeff.	SE	95% CI	ab	Total
DUET vs. WLC (X)		a	0.05	0.03	−0.02, 0.11	c’	3.42	2.92	−1.99, 9.06	0.53 (0.60) [−0.19, 2.27]	3.95 (2.89) [−1.03, 9.57]
Dietary Self-Efficacy (M)			-	-	-	b	10.87	8.09	−6.72, 27.38		
Constant		i_M_^1^	0.34	0.13	0.06, 0.58	i_Y_^2^	33.28	10.17	11.73, 52.96		
			R^2^ = 0.344, *p* =< 0.001				R^2^ = 0.117, *p* = 0.181				
			Dietary Self-Efficacy (M)				Calories (Y)			−1.30 (1.61) [−6.38, 0.40]	−12.88 (8.42) [−30.01, 1.80]
DUET vs. WLC (X)		A	0.04	0.03	−0.03, 0.10	c’	−11.58	8.13	−27.84, 2.44		
Dietary Self-Efficacy (M)			-	-	-	b	−36.45	20.42	−75.04, 3.06		
Constant		i_M_	0.43	0.14	0.15, 0.69	i_Y_	110.88	38.23	34.46, 179.19		
			R^2^ = 0.360, *p* =< 0.001				R^2^ = 0.485, *p* =< 0.001				
			Exercise Self-Efficacy (M)				Self-report MVPA (Y)			0.06 (0.11) [−0.04, 0.55]	0.89 (0.39) [0.15, 1.71]
DUET vs. WLC (X)		A	0.04	0.05	−0.07, 0.14	c’	0.83	0.39	0.11, 1.67		
Exercise Self-Efficacy (M)			-	-	-	b	1.32	1.00	−0.58, 3.30		
Constant		i_M_	0.16	0.13	−0.10, 0.41	i_Y_	−0.46	1.29	−3.24, 1.87		
			R^2^ = 0.199, *p* = 0.032				R^2^ = 0.336, *p* =< 0.001				
			Exercise Self-Efficacy (M)				Accelerometer MVPA (Y)				
DUET vs. WLC (X)		A	0.04	0.06	−0.07, 0.15	c’	0.32	0.40	−0.60, 0.99	0.05 (0.08) [−0.04, 0.32]	0.37 (0.40) [−0.59, 1.07]
Exercise Self-Efficacy (M)			-	-	-	b	1.10	0.77	−0.43, 2.49		
Constant		i_M_	0.18	0.19	−0.20, 0.56	i_Y_	1.12	1.40	−1.49, 4.14		
			R^2^ = 0.230, *p* = 0.022				R^2^ = 0.356, *p* =< 0.001				
			Dietary Self-Efficacy (M)				Weight (Y)				
DUET vs. WLC (X)		A	0.06	0.04	−0.01, 0.13	c’	−2.19	1.98	−6.18, 1.43	−0.74 (0.76) [−3.26, 0.11]	−2.93 (1.89) [−6.54, 0.36]
Dietary Self-Efficacy (M)			-	-	-	b	−13.37	7.95	−27.42, 4.67		
Constant		i_M_	0.12	0.19	−0.23, 0.49	i_Y_	10.66	8.23	−6.18, 27.18		
			R^2^ = 0.345, *p* =< 0.001				R^2^ = 0.949, *p* =< 0.001				
			Dietary Self-Efficacy (M)				Waist Circumference (Y)				
DUET vs. WLC (X)		A	0.05	0.03	−0.01, 0.13	c’	−0.92	1.18	−3.51, 1.16	−0.24 (0.31) [−1.26, 0.05]	−1.16 (1.11) [−3.50, 0.95]
Dietary Self-Efficacy (M)			-	-	-	b	−4.62	3.66	−11.83, 1.79		
Constant		i_M_	0.13	0.22	−0.25, 0.58	i_Y_	11.67	8.10	−5.08, 26.37		
			R^2^ = 0.340, *p* =< 0.001				R^2^ = 0.849, *p* =< 0.001				

^1^ i_M_ = intercept; ^2^ i_Y_ = intercept.

## Data Availability

The data presented in this study are available on request from the corresponding author.

## Supplementary Materials

The following supporting information can be downloaded at https://www.mdpi.com/article/10.3390/nu15234918/s1, Table S1: DUET intervention components that target the hypothesized Social Cognitive Theory (SCT) mediators and the specified Behavior Change Techniques (BCTs).
