# Supplementary material for: Dietary Barriers Appear to Influence the Effects of a Dyadic Web-Based Lifestyle Intervention on Caloric Intake and Adiposity: A Mediation Analysis of the DUET Trial
Source: Nutrients. 2023 Nov 25;15(23):4918. doi: 10.3390/nu15234918 (PMC10708365; doi:10.3390/nu15234918)
Supplement: Supplementary file 1 [file nutrients-15-04918-s001.zip › Supplementary_Table S1_Revisions-2701901_FINAL.pdf]

Supplementary Table S1: DUET intervention components that target the hypothesized Social Cognitive Theory (SCT) mediators and the specified Behavior Change Techniques (BCTs).

**DUET Hypothesized Mediators**	**How the DUET intervention targeted constructs of SCT**
Self-Efficacy for diet and exercise	1) The DUET website delivers weekly interactive e-Learning sessions on topics such as eating better and exercising, setting S.M.A.R.T. goals, problem solving strategies, managing stress, and overcoming barriers. The website also generates personalized progress reports to track diet, exercise, and weight and provides additional informational tools such as tracking forms, online calculators, tailored menu plans, tip sheets for grocery lists and exercise regimens. The "News You Can Use" component on the website provides critiques of current news stories in relation to guidelines. These website features provide information, strategies, and resources to achieve diet and physical activity (PA) behaviors which may enhance self-efficacy over time. [BCT# 1-31,33-36,38-40]<br>2) The interactive video sessions may also help enhance self-efficacy among survivors and partners through role modeling. [BCT# 3,4, 22,30]<br>3) Self-monitoring technologies such as Inspire Fitbit® fitness tracker and Aria 2 digital scale enhance confidence in performing daily PA through monitoring and visualizing progress over time. [BCT# 14,16,17]
Social Support for diet and exercise	1) Social support was bolstered by a buddy-system approach that counsels both survivors and their partners on strategies to request and provide support to one another in order to achieve healthy eating and daily PA goals. Survivors and partners are advised to work together to generate a shared vision and common goals towards improving their diet and PA behaviors. [BCT# 28-30]<br>2) Weekly sessions include guidance on harnessing social support to make diet and PA changes. For example, both survivors and partners received a similar interactive website session that detailed the key types of social support (i.e., emotional, informational, appraisal, and instrumental); the session underscored, largely through role modeling, various opportunities of offering, as well as asking for support. Likewise, both members of the dyad received an assignment, i.e., a weekly challenge, to practice these concepts, with the accompanying text messages for that week also reinforcing this content. [BCT# 29]<br>3) The weekly SMS text messages delivered supportive messages to promote healthy eating and exercise as a team. [BCT# 14,29,30]
Barriers for diet and exercise	1) DUET minimizes diet and exercise barriers by providing scientific-based information through website sessions on diet and PA recommendations for cancer survivors. Providing basic knowledge of dietary and PA guidelines is an important first step towards reducing barriers related to diet and PA. [BCT# 1,2]<br>2) The interactive sessions on the website also include specific diet related sessions on setting goals to limit sugar, benefits of limiting red and processed meats, increasing vegetable and fruit and whole grain intake, determining, and tracking portion sizes, and managing tempting situations. Moreover, the sessions also include information on weight loss and exercise such as how to safely lose weight and achieve 150 mins of exercise, generating safe exercise plans, and

	importance of resistance training. These practical strategies aid in overcoming barriers to diet and exercise. [BCT# 1,2,8,20,21]<br>3) The DUET website also covers sessions on commonly encountered barriers to lifestyle changes reported among cancer survivors such as managing and recognizing fatigue, stress, time, and financial constraints, managing hunger, and strategies to tackle lack of healthy foods at home. [BCT# 1,2,8,20,21]<br>4) The “Healthy Weight”, “Healthy Eating”, “Exercise”, and “Tools” features provide a variety of resources such as body mass index (BMI) and calorie calculators, exchange lists, sample meal plans, serving size handouts, supermarket survival strategies, sample exercise plans, and exercise logs, all of which reduce diet and exercise barriers by providing answers to common questions about weight management. [BCT# 1,2,20,21]
